# P-1598. Unseen but Present: Asymptomatic COVID-19 Cases and Air Travel to Hong Kong

**DOI:** 10.1093/ofid/ofaf695.1777

**Published:** 2026-01-11

**Authors:** Dylan G Ratnarajah, Weijun Yu

**Affiliations:** Georgetown University School of Medicine, Washington, District of Columbia; Georgetown University, Washington, District of Columbia

## Abstract

**Background:**

The global spread of infectious diseases was significantly influenced by the dynamics of human movement patterns, particularly for highly transmissible infections such as the SARS-CoV-2 virus, which causes COVID-19. Asymptomatic COVID-19 cases did not exhibit symptoms prior to diagnosis, making them a potential challenge for containment. Understanding air travel patterns among asymptomatic individuals was crucial for controlling the spread of the virus, however, their contribution has been understudied.

**Methods:**

Through a retrospective cross-sectional study, we analyzed 11,775 COVID-19 cases in Hong Kong. Log-binomial regression models were used to estimate the association between asymptomatic status and inbound air travel behavior 14 days prior to COVID-19 diagnosis. The median flight duration between asymptomatic and symptomatic cases was compared using the Wilcoxon rank-sum test.

**Results:**

Approximately two-thirds of the COVID-19 cases with inbound air travel history 14 days prior to diagnosis were asymptomatic. We found a significant association between COVID-19 asymptomatic status and inbound air travel behavior, which was modified by cases’ occupation categories (p< 0.0001). The prevalence of inbound air travel was significantly higher among asymptomatic cases in airport or flight crew compared to symptomatic crew members (adjusted prevalence risk ratio: 10, 95% CI: 4-20), while the prevalence of inbound air travel in asymptomatic cases outside the airport or flight crew group was 1.14 times higher than symptomatic cases (95% CI: 1.12-1.16). The median flight duration of asymptomatic cases was 4.6 person-hours shorter compared to symptomatic counterparts (p< 0.01).

**Conclusion:**

Asymptomatic COVID-19 cases indicated significantly higher prevalence of air travel within 14 days before diagnosis, suggesting potential under-detection during early travel restrictions. Asymptomatic airport and flight crew showed markedly higher travel prevalence than symptomatic counterparts, pointing to potential workplace transmission risks. These findings reveal limitations of symptom-based screening and highlight the need for proactive, symptom-independent public health strategies in future airborne disease outbreaks.

**Disclosures:**

All Authors: No reported disclosures

